# Recommendations for the ethical guidelines for publication of scientific studies: The responsibilities of editors, reviewers and the authors

**DOI:** 10.1016/j.amsu.2021.103047

**Published:** 2021-11-12

**Authors:** Sami Akbulut, Tevfik Tolga Sahin

**Affiliations:** Department of Surgery and Liver Transplant Institute, Inonu University Faculty of Medicine, 244280, Malatya, Turkey

**Keywords:** Hydatid disease, Preoperative assessment, Postoperative management, Follow up, Surgeon's responsibility, Anesthesiologist's responsibility

## Abstract

**Objective:**

We aimed to evaluate the role of anesthesiologist in the management of hydatid disease from the perspective of the editors, reviewers and the authors.

**Methods:**

We searched the PubMed/Medline database using the following keywords: (hydatid* OR echinococc*) AND (disease OR cyst) AND (anesthesiology). We have evaluated the authors, their institutions and department, and the aim of the studies. We also evaluated the studies published by anesthesiologists in terms of content.

**Results:**

The literature search showed 6344 articles published between February 2010 to 2021. Sixty-three had at least one anesthesiologist in the author list. Anesthesiologists were leading authors in 35 studies; and in 19 of them, all the authors were anesthesiologist. Sixteen (84.2%) of these articles defined the outcomes of surgical therapy and there was no information regarding anesthesia technique.

**Conclusion:**

The results of our study emphasize an important controversy regarding jurisdiction of different departments in terms of scientific research ethics. We believe that different disciplines can work together to evaluate a scientific problem and can publish a study in collaboration. But collaboration is very important and violating the subject of another field without collaboration is a deontological problem.

## Highlights

• We emphasize an important dispute regarding jurisdiction of different departments in terms of scientific research ethics.
• Different disciplines can work together to evaluate a scientific problem and can publish a study in collaboration.
• Violation of a subject of another field without collaboration is a deontological problem.

## 1 Introduction

Hydatid disease (HD) is caused by the echinococcus who belong to the taeniidae family of the cestode genus. It is zoonotic disease that causes significant morbidity in human hosts which are accidental hosts during the life cycle of the parasite. There are four type of echinococcus that cause HD in the humans [1]. The most common echinococcal parasites that cause HD in humans are E. Granulosus, which causes cystic echinococcosis (95%; HC), and E.Multilocularis, which causes alveolar echinococcosis [1]. Usually, humans are not present in the normal life cycle of this parasite and humans are accidentally infected through the ingestion of the parasite eggs through the contaminated food [1]. Therefore, this disease is a very common public health problem in countries where pastoral life and people dealing with the live stock is the primary source of house income [1]. Echinococcosis affects many organ system and tissues; but it is frequently seen in liver, lungs, spleen and kidneys [1]. The HC grow 10 mm annually according to location and it usually takes 10–15 years and this period is shorter in complicated cysts [2]. Clinical signs and symptoms usually develop as a result of compression of the neighboring tissue and organ, rupture to the biliary and bronchial tract, compression of the vascular structures and perforation [3].

Anamnesis, physical examination, radiological tools, and serologic tests are evaluation as a whole for the diagnosis of the HD. During the diagnostic process, general practitioner, internists (including the gastroenterologists), infectious disease specialist, thoracic and general surgeons may be involved. All though there is some variation among different specialties, the treatment modality for HD is directed according to the classification of Gharbi and World Health Organization Informal Working Group on Echinococcosis (WHO-IWGE) which provided some standardization for the treatment of this disease [4]. The treatment alternatives vary a lot but mainly include a combination of watch and wait, anti-helminthic, interventional radiological approaches (percutaneous aspiration-injection-reaspiration [PAIR] etc), open or laparoscopic surgery [4,5]. The surgeon and the interventional radiologist in charge decide the best option for the treatment for each patient.

The diagnosis, treatment and postoperative follow-up of the patients are directed according to a certain protocol and postoperative follow-up is almost always directed by the primary consulting surgeon of the patients. Anesthesiologists, immunologists and parasitologists do not take part in the clinical management of HD. Parasitologists and immunologists play an important role in the both developing experimental models for behavior of the parasites and immunodiagnostics [6,7]. The public health professionals have a role in epidemiologic studies for the global distribution of the HD [8]. Anesthesiologists take part in preoperative evaluation of the patients and intraoperative management of the patients during surgery. For patients who require postoperative ICU care, attending surgeon and the intensivists are involved together. Globally, anesthesiologists taking part in long-term follow-up of the HD patients in postoperative period is not a common practice because it is not in the practice field of this specialty. The aim of the present study was to evaluate the role of anesthesiologists in the studies regarding HD published in the literature in a format of a literature review. Our secondary aim was to discuss the roles of editor, reviewer and the authors by analyzing a study about HD written by a team of anesthesiologists.

## 2 Materials and methods

### 2.1 Literature review

We have performed a literature review in PubMed database in a specific time period between February 2010 and 2021. The review was performed in two stages. In the first stage the key words that were used were (hydatid* OR echinococcus*) AND (disease OR cyst) AND (anesthesiology) which yielded 69 articles. In the second stage of the literature review we used the key words (hydatid* OR echinococcus*) AND (disease OR cyst) for the same time period and came up with 6629 articles. We analyzed the quality and suitability of the articles regarding our research subject. We also noted the leading author and departments of the leading and the co-authors. If the leading authors was from anesthesiology or if the team of authors of the study were from anesthesiology; then we analyzed the content of the article in terms of suitability for the area of anesthesiology.

### 2.2 The index article in question

We read the recent article published by Tercan and colleagues [9] in Rev Bras Anestesiol. The authors stated that they present the clinical features, interventional procedures, and anesthesia methods of the patients who were treated for hydatid cyst (HC). We will present out comments in the results section of this study. Part of this section has been published by Ann Med Surg (Lond) and mentioned article published in both English and Portuguese [10].

## 3 Results

### 3.1 Literature review

In the initial stage of the literature review we found 69 articles with the key word we have stated in the materials and methods section. Six of these studies were not related with HD. Therefore, 63 articles were included for evaluation for the present study. In the second stage of the literature review, we have found 6629 articles with the key words we have used; as we have stated in the materials and methods section of the manuscript. Two hundred and eighty-five articles were not related with HD and 6344 (including the 63 articles that included at least one anesthesiologist in the authors list) articles were included for evaluation in the present study. We performed our analysis criteria on these 6344 articles that we have found as a result of our PubMed search.

Our analysis showed that 0.99% of the studies had at least one anesthesiologist in the authors list. The analysis of the 63 articles with anesthesiologists in the authors list showed that 48 were case reports and 15 were original research articles. Sixty-one articles were clinical studies and 2 were experimental studies. The top four countries of origin of these studies were Turkey (n = 16), India (n = 14), China (n = 10), and Iran (n = 5). Thirty-five articles included two or more anesthetists on the list of authors. Anesthesiologists were leading authors in 35 studies; and in 19 articles all the authors were from the department of anesthesiology. Sixteen of these 19 articles defined the outcomes of surgical therapy and in six of these articles, there is no information regarding routine and specific techniques applied by anesthesiologists. In 17 of the 19 articles, there is no surgeon on the author list despite the description of the results of the surgical therapy. We considered these as deontological and ethical problem in terms principles of scientific research. Characteristics of 63 articles in which anesthetists are among the authors are summarized in Table 1.

**Table 1:** Main features of hydatid disease studies that contained anesthesiologists in the author list (PubMed study period: 03.02.2010 and 03.02.2021).

References	PMID	Years	Country	Journal Name	Article Type	Total Author	First Author Clinic	Order of Anesthesio	Main subject of the article
Mrzljak	33210197	2021	Croatia	Parasitol Res	Case Rep	6	Medicine	5	Liver graft harbouring HC
Yimamu	33308341	2020	China	Cardiol Young	Case Rep	3	Cardiac Surgery	2	Managemetn of primary pericardial HC
Savu	33121083	2020	Romania	Medicina (Kaunas)	Case Rep	12	Thoracic Surgery	10	Primary pleural hydatidosis
Gupta	33013101	2020	India	J Emerg Trauma Shock	Case Rep	3	Anesthesiology	All	Anaphylactic shock due to HC rupture
Maghrebi	33011658	2020	Tunisia	Int J Surg Case Rep	Case Rep	10	General Surgery	8	Retroperitoneal HC rupture
Viderman	33004207	2020	Kazakhstan	Rev Bras Anestesiol	Case Rep	6	Neurosurgery	2-3-5-6	Complicated HC in spinal cord
Hamza	32777766	2020	Kosova	Int J Surg Case Rep	Case Rep	5	General Surgery	3	ERCP for jaundice caused by HC
Azulay	32158277	2020	Israel	Int J Surg Case Rep	Case Rep	5	Thoracic Surgery	5	Management of huge pulmonary HC
Tercan	32532549	2020	Turkey	Rev Bras Anestesiol	Original	4	Anesthesiology	All	Management of 393 patients with HC
Erdoğan	32082892	2019	Turkey	Turk Gogus Kalp Dam.	Case Rep	6	Thoracic Surgery	4	Treatment of HD infiltrating into myocardium
Wu	31852095	2019	China	Medicine (Baltimore)	Case Rep	3	Anesthesiology	All	Arndt endobronchial blocker usage for patient with HD
Kuzmanovska	31819311	2019	Macedonia	Med Arch	Case Rep	8	Anesthesiology	1-2-6-7	Neurologic complications after liver HC surgery
Seyedsadegh	31673270	2019	Iran	Iran J Parasitol.	Case Rep	4	General Surgery	3	Gluteal HC
Pencovich	28870127	2019	Israel	Matern Fetal Neo Med	Case Rep	8	General Surgery	4	HC surgery during pregnancy
Panteleev	31846993	2019	Russia	Infez Med	Original	7	General Surgery	6	Surgery for liver cystic and alveolar echinococcosis
Apaydın	30377526	2018	Turkey	Ann Med Surg (Lond)	Case Rep	2	Thoracic Surgery	2	Giant pulmoner HC
Apaydın	30118962	2018	Turkey	Int J Surg Case Rep	Case Rep	2	Thoracic Surgery	2	Pulmoner HC presenting as plevral effusion
Aydin	29750934	2018	Turkey	Ann Thorac Surg	Case Rep	4	Thoracic Surgery	2	Laparotomy for pulmoner HC: New technique
Su	30032679	2018	China	J Int Med Res	Original	6	Anesthesiology	1-3--4-5-6	TAP block for postoperative pain in patients with HC
Fallah	29307164	2017	Iran	Acta Med Iran	Case Rep	3	Anesthesiology	1	Abdominal primary disseminated HC
Eberl	28978605	2017	Netherlands	BMJ Case Rep	Case Rep	4	Anesthesiology	All	Anaphylactic shock due to HC rupture during PAIR
Baradan	28761474	2017	Iran	Iran J Parasitol	Case Rep	3	General Surgery	3	Cerebral HC
Abad-Torrent	28183584	2017	Spain	J Clin Anesth	Case Rep	5	Anesthesiology	1–5	Monitoring intraoperative analgesis by pupillometry
Naldan	27793342	2017	Turkey	Surgery	Case Rep	5	Anesthesiology	1 and 2	Extrapelvic HC presenting as perianal abscess
Yang	29078881	2017	China	J Surg Res	Original	6	Anesthesiology	1-2-4-5-6	Enhanced Recovery after Surgery in Alveolar echinococc.
Fallah	28979357	2017	Iran	Iran J Parasitol	Original	3	Anesthesiology	1	Prelalence of human hydatidosis
Li	28678921	2017	China	Braz J Med Biol Res	Original	5	Anesthesiology	1-2-4-5	IgE and IgG1 in HC fluid
Maitra	27687398	2016	India	J Clin Anesth	Case Rep	4	Anesthesiology	All	Reexpansion pulmonary edema after HC excision
Atalan	27551181	2016	Turkey	Eurasian J Med	Case Rep	5	Anesthesiology	1–4	HD presenting as Cerebral and Spinal Intradural Metastases
Mirijello	27516403	2016	Italy	J Emerg Med	Case Rep	6	Emergency Med.	6	Anaphylactic reaction due to cardiac and hepatic HC
Ye	26711523	2016	China	Am J Trop Med Hyg	Case Rep	4	Anesthesiology	All	Immunol characteristics of recurrent HC-induced anaphylactic
Ye	28095662	2016	China	Korean J Parasitol	Original	7	Anesthesiology	All	Perioperative anaphylactic shock due to HC rupture
Sarmast	27904571	2016	India	J Res Med Sci	Original	7	Neurosurgery	3	Hydatid disease everywhere
Zhang	26968945	2016	China	BMC Immunol	Original	3	Anesthesiology	All	Effect of dexamethasone on HC-induced allergic react (Exper)
Zhang	26603168	2016	China	Immunol Res	Original	6	Anesthesiology	All	Immune tolerance in mice with anaph. shock after HC (Exper)
Karuppiah	26755844	2015	India	Indian J Anaesth	Case Rep	4	Anesthesiology	All	Role of transesophageal ECHO on cardiac HC
Jain	26139754	2015	India	Ann Card Anaesth	Case Rep	5	Cardiac Surgery	1-2-4	Cardiac HC and transesophagial ECHO
Hela	26130458	2015	Tunisia	Libyan J Med	Case Rep	8	Cardiac Surgery	5	Cardiac HC presenting with lower limb ischemia
Zeren	26069180	2015	Turkey	Acta Med Iran	Case Rep	3	General Surgery	3	Gluteal HC
Kaur	25684819	2015	India	Indian J Anaesth	Case Rep	4	Anesthesiology	1–22	Intraoperative airway obstruction due to ruptured HC
Hela	28349810	2015	Tunisia	Libyan J Med.	Case Rep	8	Thoracic Surgery	5	Cardiac HC presenting with lower limb ischemia
Richter	25547080	2015	Germany	Parasitol Res	Case Rep	7	Tropical Med	2	Anaphylactic shock due to therapeutic puncture of HC
Aydinli	26074280	2015	Turkey	Liver Transpl	Original	8	General Surgery	6	Liver transplantation for alveolar echinococcosis
Bartin	26170748	2014	Turkey	Ulus Cerrahi Derg	Case Rep	5	General Surgery	4–55	HC of the thyroid gland
Davarci	25781300	2014	Turkey	West Indian Med J	Case Rep	5	Anesthesiology	1-2-3-5	Anaphylactic shock due to nonruptured HC
Kumar	25584268	2014	India	J Clin Diagn Res	Case Rep	5	General Surgery	2-3-5	Intraperitoneal rupture of HC
Marashi	25237636	2014	Iran	Anesth Pain Med	Case Rep	4	Anesthesiology	All	Anaphylactic shock due to pulmonary HC
Demir	25229301	2014	Turkey	Rev Soc Bras Med Tro	Case Rep	3	Anesthesiology	1	Primary HC of the soft tissue
Bostan	25125959	2014	Turkey	Hippokratia	Case Rep	5	Forensic Med	5	Forgetting instrument in the abdominal cavity during HC surgery
Suner	24890769	2014	Turkey	Rev Port Cardiol	Case Rep	8	Cardiology	7	HC compressing the pulmonay artery and atrial septal defect
Ansari	24631343	2014	India	J Infect Public Health	Case Rep	6	General Surgery	2	Breast HC
Altay	24770834	2014	Turkey	Clin Ter	Original	6	Anesthesiology	All	Anestetiz management in HC
Dogra	24027375	2013	India	J Cardiovasc Dis Res	Case Rep	3	Anesthesiology	All	Perioperative transesophagial ECHO for isolated pericardial HC
Benali Zel	24847399	2013	Morocco	Pan Afr Med J	Case Rep	1	Anesthesiology	All	Pulmonary HC in a child
Bavullu	27366371	2013	Turkey	Turk J Anaesthesiol Reanim	Original	5	Anesthesiology	1-2-3-4	Two different sedative drugs for percutaneous drainage of HC
Bensghir	22754444	2012	Morocco	Saudi J Anaesth	Case Rep	7	Anesthesiology	All	Anaphylactic shock during HC surgery
Hariharan	24765449	2012	India	Clin Pract	Case Rep	5	Anesthesiology	All	Excision of intracranial HC
Bharati	24027387	2012	India	Niger J Surg	Case Rep	2	Anesthesiology	1	Primary HC in gastrocnemius muscle
Khanna	21373723	2011	India	Singapore Med J	Case Rep	3	Anesthesiology	All	Anaphylactic shock after HC surgery
Bajwa	25885312	2011	India	Anesth Essays Res	Case Rep	5	Anesthesiology	1-2-4-5	Anestetic challenges in pulmonary and hepatic HC
Lukmanova	22308705	2011	Russia	Med Parazitol (Mosk)	Original	4	NA	NA	Distribution of HLA specificity in patients with HC
Li	21896803	2011	China	Am J Trop Med Hyg	Original	5	Anesthesiology	All	Anaphylactic shock after HC surgery
Tomar	23189891	2010	India	Indian Heart J	Case Rep	5	Anesthesiology	All	Interatrial septum HC removed under cardiopulmonary bypass

### 3.2 Comment on index article

This article is about abdomino-thoracic surgery of HC but all of the authors are anesthesiologists. Almost entire article is about the demographic and clinical characteristics of the HC and only a limited information regarding the anesthesia methods of the patients is provided. In fact, the anesthesia methods they mentioned are not related to HC and are a standard procedure applied to all surgical procedures performed with general anesthesia. Publication of this article without the consent of the primary surgeons responsible for patients is an major deontological problem.

The authors state that 50 HD patients (12.9%) in the study were operated due to spontaneous HC rupture. Although we performed a thorough literature search, we have not come across any study reporting such high spontaneous HC rupture rates. The authors should state how they managed patients undergoing surgery for HC rupture. They should have indicated which doses of steroid and antihistaminic medication they were given to these patients. They should give information about the extubation of patients with HC rupture and their early postoperative follow-up. If they had highlighted these issues, than this article would have been related with the anesthesiologists.

The authors state that 60 patients underwent PAIR and 30 patients underwent laparoscopic surgery which is comparable to the best-case series reported in the literature. The authors should explain whether patients who underwent PAIR developed any complications during the procedure and whether they used a specific anesthesia method for PAIR. In fact, the subject of PAIR is a topic that radiologists should present with their technical details. As a matter of fact, all of the studies published on this subject in the literature were written by radiologists and primary responsible clinicians. Similarly, the authors should have explained which anesthesia method they used during laparoscopic HC surgery, what levels were abdominal pressure maintained, and whether pulmonary complications developed after laparoscopy. If they had highlighted these issues, it was understandable that the article could have been related to anesthesiologists.

The authors have summarized their results regarding intraoperative complications and postoperative recurrences in Table- 4. The postoperative recurrences and intraoperative complications are under the responsibility of the treating surgeons. We believe that it is not the responsibility of the anesthesiologist to follow the patients for the recurrence of a particular disease.

This study includes a cohort of about 400 patients who received HC surgery. The authors should have provided information regarding the adjuvant and neoadjuvant albendazole treatment which is the usual procedure in studies of this kind. For example, in the present study, the duration and type of adjuvant therapy in abdominal and thoracic HCs should have been stated. Furthermore, the authors should clarify whether they have used neoadjuvant anti-helminthic therapy was used in pulmonary HCs and if they have used such a treatment, they should state if they have encountered any HC perforation as a result of neoadjuvant albendazole treatment. Another point that needs emphasis is related with the complication rates following the pulmonary HC because the current literature suggests that pulmonary HC have higher complication rates following any operative intervention. However, in the present study, 82 patients were operated due to pulmonary HC but no complication was reported which is not consistent with the current knowledge.

Another point that should be emphasized is related with the modality of therapy that is applied. There is no information regarding the radical and conservative surgeries, the success rate of PAIR procedure, the biliary complication rates and the necessity of ERCP related with these complications. In addition, detailed information is needed regarding the recurrence rates following surgery for ruptured HCs. In brief, at least five different studies on very different topics such as pulmonary HCs, HC perforation, laparoscopic management of HCs, PAIR for HC, factors affecting postoperative biliary fistula can be prepared from the cohort of the present study; however, the authors have included these wide variety of patients in a single study and did not provide crucial information that would guide other researchers. This is mainly due to the fact that all the authors are anesthesiologists who are not experienced in the management of HCs in terms of clinical perspective.

More than 80% of the article word count is related with the demographic and clinical features of HC disease, and all the authors are anesthesiologists which is a serious deontological. In our opinion, there is no difference between hydatid cyst’ patients and other patients from the perspective of anesthesiologic management. The risk of allergic reaction may be a prominent factor that would be important for anesthesiologists and this complication was very rare in the present study.

To sum up, we do not make an argument among the authors of this study that anesthesiologists should not be included. We emphasize that the absence of surgeons and radiologists among the authors of the study is deontologically unacceptable.

## 4 Discussion

The results of our study emphasize an important controversy regarding jurisdiction of different departments in terms of scientific research ethics. The criteria for authorship in an article is already defined by the international committee of medical journal editors and their recommendations clearly state that '' *In addition to being accountable for the parts of the work he or she has done, an author should be able to identify which co-authors are responsible for specific other parts of the work. In addition, authors should have confidence in the integrity of the contributions of their co-authors. All those designated as authors should meet all four criteria for authorship* '' [11]. The authors of the index study do not meet this criteria.

We believe that different disciplines can work together to evaluate a scientific problem and can publish a study in collaboration. But collaboration is very important and violating the subject of another field without collaboration is a deontological problem. Therefore, in the discussion section of our study, we would like to give our recommendations for authors, editors and the reviewers in order to prevent such mistakes in future.

## 5 Recommendations

### 5.1 For editors

The editors should evaluate the heading and the abstract section of the manuscript very carefully. They should also evaluate the departments of the authors in the author list. The editors are responsible for the suitability of the content of the study and the specialty area of the authors. The editors should collaborate with reviewers that prioritizes academic quality of studies and should include them in the editorial board of the study. Another option is to compartmentalize journals and become highly specific for content of the studies.

The editors should evaluate the statistical methods of the clinical ad experimental studies that are submitted. One way to overcome the workload under these circumstances is to have statisticians in the editorial board of the journal. In necessary situations, the data set of the authors should be requested, and the analysis of the data set should be controlled.

The email address and the ORCID numbers of the authors in the study should be checked by the editors. Each author should send a copy write transfer form and should send it from his/her own e-amil address. The editors should also check the institutional review board (IRB) approval including the date and approval number. The IRB approval number and date should be stated in the manuscript clearly. Furthermore, the copy of the IRB approval should be sent to the journal.

Applying deontological and ethical rules for the studies being evaluated for publication is especially true for journals with publication fees. These journals should never violate ethical principles. In our opinion, a reasonable amount of publication fee can be requested but this should never affect the publication process of the journal. One strategy tom overcome such a problem is to standardize the evaluation process of the journal and transparency of the evaluation process. This means that authors of a particular study that is being evaluated for publication should be able to see the reviewer (anonymously) comments online in any time period. This is a very ideal strategy for overcoming any questions regarding the publication process of the journal.

The editors can increase the reviewer pool by getting suggestions from the authors. However, the evaluation process could be biased and therefore, the suggested reviewers should not be more than one third of the reviewers for a specific study being evaluated.

The editors should be open to criticisms regarding any published study in their journal. Every criticism should be evaluated in the commission and necessary corrections should be requested from the authors of the study if the criticisms are valid. The response of the authors should always be published. Retraction of studies with major mistakes is a valid option and editors should not hesitate. However, it is common fact that most of the editors are immune against any deontological and ethical problems and they usually do not accept the consequences of their actions.

Therefore, there should be an international and national review board for evaluation of such violations and certain penalties should be applied to the publishing group, journal and the editor in chief. National board should operate under the criteria determined by the supreme education council of individual country and also according to the criteria of the international review board. The international board should be formed from the most prestigious and high impact researchers of every country and a consensus statement and regulations should be prepared. Violation of these regulations should be subject to penalty. Globally, there are more than 100 countries and more than 10.000 journal. For practical purposes the national and international review boards should collaborate in order to provide a better service. Website, a definitive address and a legitimate license should be obtained for every journal. The website should be active and should always have an English option even if publication language is different.

### 5.2 For authors

Similar procedures should be applied for the authors. Forgery and falsification should be subject to penalty according to the legal structure of the country of the authors. The authors should have a responsibility for their action, and they should be responsible to the same institutions as the journals and the publishing groups. This would persuade the authors to be more careful about study design. These precautions could prevent the ethical violations observed in the index study that we have commented about. As we have presented in our study there are two articles that are published that only have anesthesiologists in the author list, but the study is reporting results of surgical therapy for hydatid disease [9,12]. One other point that needs emphasis is the fact that none of the surgeons in the institutions from which these studies were performed did not object to this problem.

### 5.3 For reviewers

The reviewers should be blinded against the country and the authors of the study that is submitted for evaluation. Furthermore, the reviewers should not have access to each other's comments. This will minimize the bias during the evaluation process. The editors have the primary responsibility regarding this issue. The reviewers have the right to decline a reviewer request, if the submitted manuscript is out of the scope of their specialty. This is the most appropriate deontological approach. The editors should evaluate the reviewers for their specialty and area of interest in their research. The articles should be sent according to the area of interest of the reviewers.

## Ethical approval

None. Our paper is in the format of literature review.

## Funding

This literature review did not receive any specific grant from funding agencies in the public, commercial, or not-for-profit sectors.

## Author contribution

Sami Akbulut and Tevfik Tolga Sahin: wrote the manuscript. Sami Akbulut and Tevfik Tolga Sahin: Supervised the writing process and revised the manuscript.

## Consent

This paper prepared as letter to the editor. Patients data were not used in this study. Therefore concent approval is not required.

## Research registration

Not Applicable.

## Registration of Research Studies

1.Name of the registry: Not Applicable. Because this study is prepared as letter to the editor (comment)
2.Unique Identifying number or registration ID:
3.Hyperlink to your specific registration (must be publicly accessible and will be checked):

## Guarantor

Prof. Sami Akbulut, and Prof. Tevfik Tolga Sahin, are the guarantors for the present commentary and they take full responsibility for the comments and the auxiliary data presented in the commentary article.

## Declaration of competing interest

The authors stated that they have no conflict of interest.
